# Hémothorax spontané révélant une vascularite de Wegener chez une femme enceinte

**DOI:** 10.11604/pamj.2016.25.35.10418

**Published:** 2016-09-28

**Authors:** Hind Serhane, Msougar Yassine, Lamyae Amro

**Affiliations:** 1 Service de Pneumologie, Hôpital Arrazi, CHU Mohamed VI, Laboratoire PCIM, FMPM, Université Cadi Ayyad, Marrakech, Maroc; 2 Service de Chirurgie Thoracique, Hôpital Arrazi, CHU Mohamed VI, Laboratoire PCIM, FMPM, Université Cadi Ayyad, Marrakech, Maroc

**Keywords:** Hémothorax spontané, vascularite, thoracotomie, Spontaneous hemothorax, vasculitis, thoracotomy

## Abstract

L’hémothorax spontané demeure une pathologie rare. Ses étiologies sont multiples mais restent parfois inconnues. Chez certains patients, la thoracotomie peut être le seul recours pour déterminer son origine. Les vascularites n’ont pas été rapporté comme cause habituelle des hémothorax spontanés. La grossesse ne semble pas avoir d’effet causal, ni aggravant des hémothorax spontanés, ni des vascularites. Nous rapportons une observation assez particulière d’une jeune patiente, présentant au cours de sa grossesse un hémothorax spontané secondaire à une vasularite de type Wegener, diagnostiquée par la biopsie pleurale faite au cours d’une thoracotomie exploratrice et confirmée par le dosage des ANCA.

## Abstract (en)

Spontaneous hemothorax is a rare condition. Its causes are multiple but sometimes they remain unknown. In some patients, thoracotomy may be the only means to determine hemothorax origin. Vasculitis have not been reported as a common cause of spontaneous hemothorax. Pregnancy does not appear to have causal or aggravating effect on spontaneous hemothorax or on vasculitis. We here report the peculiar case of a young patient presenting during pregnancy with spontaneous hemothorax secondary to Wegener’s vasculitis. The latter was diagnosed by pleural biopsy performed during exploratory thoracotomy and confirmed by ANCA assays.

## Introduction

L’hémothorax est une affection relativement fréquente, souvent associé à un traumatisme, d’origine iatrogène, à une coagulopathie, à une dissection aortique, à un infarctus pulmonaire, à une néoplasie ou un pneumothorax spontané. Par contre, l’hémothorax spontané est une pathologie rare [1], les étiologies sont multiples [2] mais demeurent parfois inconnues en vue d’une thoracotomie exploratrice [3]. Les vascularites n’ont jamais été rapportées comme éventuelle cause des hémothorax, à la limite de notre connaissance. La grossesse ne semble pas avoir d’effet causal, ni aggravant des hémothorax spontanés, ni des vascularites [4]. Nous rapportons une observation assez particulière d’une jeune patiente, présentant au cours de sa grossesse un hémothorax spontané secondaire à une vasularite de type Wegener.

## Patient et observation

**Figure 1:**
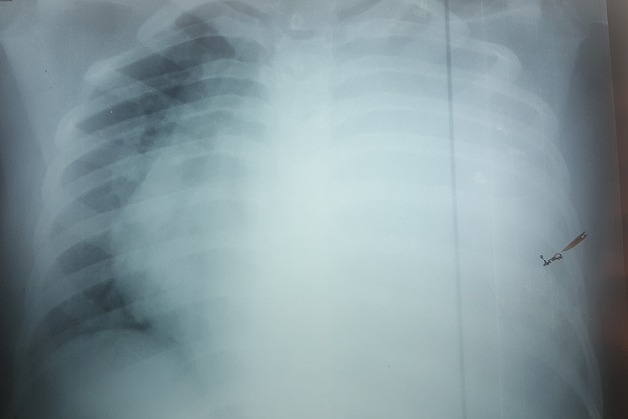
Radiographie thoracique à l’admission

**Figure 2:**
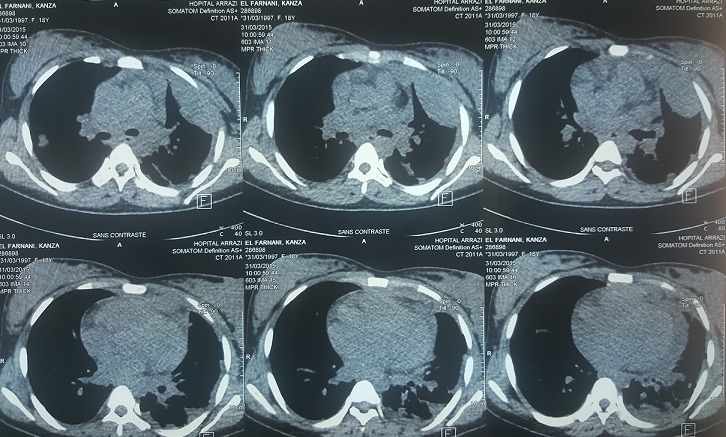
TDM thoracique

**Figure 3:**
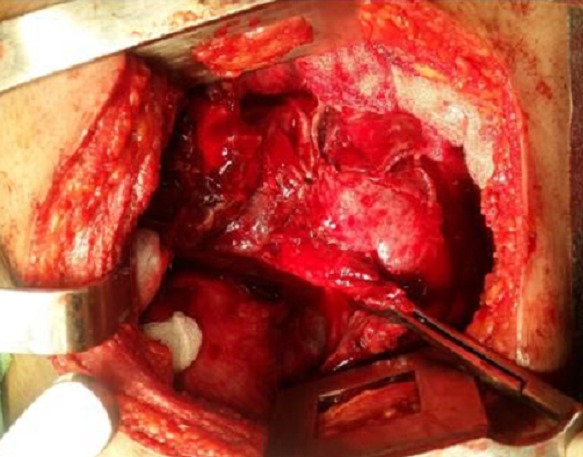
Thoracotomie exploratrice

**Figure 4:**
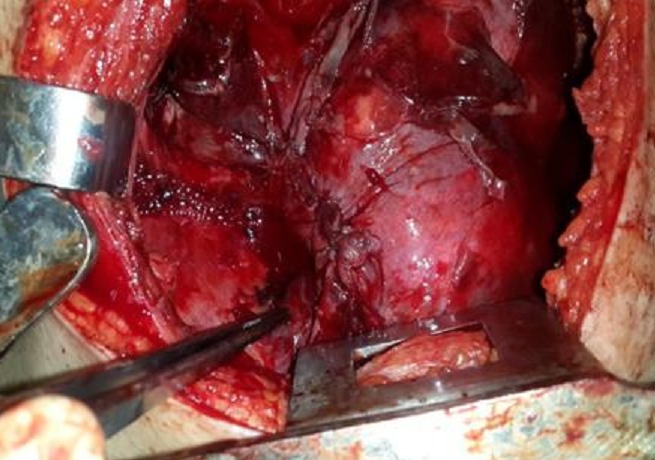
Aspect per-opératoire montrant des formations kystiques

Mme K. F âgée de 18 ans, enceinte de 26 SA, non tabagique, jamais traitée pour tuberculose, sans contage tuberculeux récent et sans notion de traumatisme thoracique. Qui a présenté une dyspnée d’installation rapidement progressive stade IV de Sadoul associée à une toux et une douleur thoracique gauche en point de côté. L’examen clinique trouvait une patiente consciente, polypneique à 32 cyles/minutes avec des signes de luttes respiratoires. L’examen pleuro-pulmonaire avait objectivé un syndrome d’épanchement liquidien de tout l’hémithorax gauche. L’examen obstétrical avait noté: une hauteur utérine à 22 cm, des bruits cardiaques foetaux à 142 battements/minutes, un bassin normal, pas de contractions utérines, un col fermé postérieur, une présentation haute et une poche des eaux intacte au toucher vaginal. La patiente a été prise en charge en milieu de réanimation. Après mise en condition de la patiente. Une radiographie thoracique, faite avec protection, avait révélé un hémithorax gauche opaque refoulant les éléments du médiastin vers le côté controlatéral (Figure 1). Une Ponction pleurale avait ramené du liquide hématique, dont l’étude cytochimique montrait un taux de protides à 40 g/L, et un taux d’hématocrite à 26.9%, le rapport hématocrite pleural/hématocrite plasmatique>50%, en faveur d’un hémothorax. L’étude bactériologique du liquide pleural n’a pas isolé de germe, la recherche de BK dans le liquide pleural était négative. Un drainage thoracique a été réalisé à l’aide d’un drain de Joly 28 G ramenant initialement 1litre de liquide hématique. L’échographie obstétricale avait montré une grossesse monofoetal évolutive, présentation transversale, liquide amniotique de quantité suffisante, placenta fundique. Après stabilisation, la patiente a été transférée au service de pneumologie pour bilan étiologique de son hémothorax. Deux ponctions biopsies pleurales objectivant des remaniements inflammatoires non spécifiques à l’étude anatomopathologique. La numération formule sanguine avait noté un taux d’hémoglobine à 6.3 g/l, nécessitant une transfusion de 6 Culots globulaires à deux reprises. Une TDM thoracique avec protection (Figure 2) avait révélée un épanchement pleural de grande abondance multicloisoné associé à des nodules intra-parenchymateux et un épanchement péricardique de faible abondance. La bronchoscopie avait montré un état inflammatoire diffus de 1er degré dont les biopsies n’avaient pas montré d’anomalie histologique. Le cytodiagnostic du liquide d’aspiration n’avait pas trouvé de cellules malignes. La ponction transpariétale scanno-guidée d’un des nodules parenchymateux n’était pas réalisable devant l’impossibilité de se mettre en décubitus dorsale par la patiente. Une alternative echoguidée a été faite mais l’étude anatomopathologique objectivait toujours un remaniement inflammatoire non spécifique. La thoracoscopie aussi a été réalisée et n’avait toujours pas abouti au diagnostic. Devant l’impasse diagnostic, le recours à la thoracotomie exploratrice (Figure 3) était indispensable et avait montré la présence de formations kystiques à parois fines disséminées à toute la cavité thoracique (Figure 4), des biopsies multiples ont été réalisé dont l’étude anatomopathologique avait finalement identifié un aspect morphologique compatible avec des lésions de vascularite étendue et nécrosante. La patiente avait accouché dans les jours qui suivent d’un bébé de sexe féminin, le bébé a était pris en charge au service de réanimation néonatale avec bonne évolution. Puis, Le reste du bilan à la recherche du type de vascularite était en faveur d’une maladie de Wegener avec des anticorps anti-cytoplasme des polynucléaires de type C (C-ANCA) positifs. Des bolus de corticothérapie et d’immunosuppresseur ont été démarrés avec une nette amélioration.

## Discussion

L’hémothorax est définit par un liquide pleural dont le taux d’hématocrite est supérieure à 50% de l’hématocrite plasmatique [2]. Il est relativement lié aux traumatismes ouverts ou fermés du thorax, aux interventions thoraciques comme les biopsies, les drainages et les cathétérismes. L’hémothorax spontané est rare [1, 2] mais constitue une véritable urgence vitale lorsqu’elle survient chez la femme enceinte [5]. Sa survenue au cours de la grossesse est inhabituelle et responsable d’une détresse respiratoire aigue, pouvant mettre en jeu le pronostic vital de la patiente et du bébé [6]. Son diagnostic étiologique est souvent difficile [5]. Plusieurs moyens diagnostiques, sont possibles mais le recours à la thoracotomie exploratrice s’avère dans certains cas indispensable pour identifier l’étiologie, ce qui était le cas de notre patiente. Devant un hémothorax spontané certains diagnostics sont à évoquer de principe, et chez la femme enceinte une pleurésie métastatique d’un choriocarcinome pulmonaire devrait être éliminée. Le Tableau 1 résume les principales étiologies des hémothorax spontanés. Très peu de données ont concerné la survenue d’hémothorax spontané secondaire à une vascularite et notamment une maladie de Wegener. Chez les femmes porteuses de cette vascularite, le risque de poussée de la maladie est de 25% au cours de la grossesse [7], mais le risque de survenue d’hémothorax n’a jamais été rapporté dans la littérature, à la limite de notre recherche bibliographique. L’hypothèse qui pourrait expliquer la survenue d’hémothorax dans le cadre d’une vascularite au cours de la grossesse, est l’état de stress vasculaire important dû aux changements hormonaux qui se produisent chez la femme enceinte [8]. De plus la possibilité de survenue d’anévrysmes vasculaires et de ruptures vasculaires augmente quand l’âge gestationnel avance. Ceci quand il survient sur un terrain vasculaire déjà fragilisé par une vascularite, pourrait entrainer plus facilement la rupture des petits vaisseaux pulmonaires, touchés par la maladie. Cette rupture quand elle se fait au niveau de la plèvre est à l’origine d’hémothorax comme dans le cas de notre observation.

**Tableau 1:** Principales étiologies des hémothorax spontanés

	Hemopneumothorax
	**Les causes vasculaires**
**1.**	Les malformations arterio-veineuses
**2.**	Les télangiectasies hémorragiques héréditaires
**3.**	Les dissections ou ruptures anévrysmales
**4.**	Le syndrome d’Ehlers Danlos
**5.**	La neurofibromatose
	**Les anomalies hématologiques**
**1.**	Les maladies hémorragiques congénitales
**2.**	Les coagulopathies acquise
**3.**	Les troubles hématopoïétiques
	**Les néoplasies**
**1.**	Les tumeurs malignes (mésothéliome, adénocarcinome, sarcome d’Ewing, sarcome…)
**2.**	Les tumeurs bénignes (thymome bénigne, hémangiome veineux …)
	**Les complications de la grossesse**
**1.**	La grossesse ectopique
**2.**	La maladie gestationnelle trophoblastique
**3.**	Hémopéritoine peripartal
**4.**	Rupture d’un hématome suprascapulaire
	**Les maladies thoraciques**
**1.**	Rupture d’adhésion pleurale
**2.**	Infarctus pulmonaire sur maladie thromboembolique
**3.**	Infection pulmonaire
**4.**	Exostose costale
**5.**	Séquestration pulmonaire
	**Complications impliquant le diaphragme**
**1.**	Endométriose
**2.**	Implantation d’une grossesse ectopique primaire
	**Complications thérapeutiques**
**1.**	Procédures de laparoscopie
**2.**	Drainage thoracique
**3.**	Biopsie pleurale
**4.**	Chirurgie gastrointestinale
	**Cause Idiopathique**

## Conclusion

Les étiologies des hémothorax spontanées sont assez exhaustives, et que le recours à la thoracotomie à visée diagnostique peut être extrêmement utile en cas d’impasse diagnostique. Celle-ci est une observation assez originale. Qui montre que devant un hémothorax spontané, le diagnostic de vascularite bien que rare, peut figurer parmi les diagnostics à évoqués.
